# Multi-site and nasal swabbing for carriage of *Staphylococcus aureus*: what does a single nose swab predict?

**DOI:** 10.1016/j.jhin.2017.01.015

**Published:** 2017-07

**Authors:** B.C. Young, A.A. Votintseva, D. Foster, H. Godwin, R.R. Miller, L.W. Anson, A.S. Walker, T.E.A. Peto, D.W. Crook, K. Knox

**Affiliations:** a Nuffield Department of Medicine, University of Oxford, Oxford, UK; b Nuffield Department of Primary Care Health Sciences, University of Oxford, Oxford, UK

**Keywords:** *Staphylococcus aureus*, Carriage, Multi-site screening, *spa* typing

## Abstract

**Background:**

Carriage of *Staphylococcus aureus* is a risk for infections. Targeted decolonization reduces postoperative infections but depends on accurate screening.

**Aim:**

To compare detection of *S. aureus* carriage in healthy individuals between anatomical sites and nurse- versus self-swabbing; also to determine whether a single nasal swab predicted carriage over four weeks.

**Methods:**

Healthy individuals were recruited via general practices. After consent, nurses performed multi-site swabbing (nose, throat, and axilla). Participants performed nasal swabbing twice-weekly for four weeks. Swabs were returned by mail and cultured for *S. aureus*. All *S. aureus* isolates underwent *spa* typing. Persistent carriage in individuals returning more than three self-swabs was defined as culture of *S. aureus* from all or all but one self-swabs.

**Findings:**

In all, 102 individuals underwent multi-site swabbing; *S. aureus* carriage was detected from at least one site from 40 individuals (39%). There was no difference between nose (29/102, 28%) and throat (28/102, 27%) isolation rates: the combination increased total detection rate by 10%. Ninety-nine patients returned any self-swab, and 96 returned more than three. Nasal carriage detection was not significantly different on nurse or self-swab [28/99 (74%) vs 26/99 (72%); χ^2^: *P* = 0.75]. Twenty-two out of 25 participants with first self-swab positive were persistent carriers and 69/71 with first self-swab negative were not, giving high positive predictive value (88%), and very high negative predictive value (97%).

**Conclusion:**

Nasal swabs detected the majority of carriage; throat swabs increased detection by 10%. Self-taken nasal swabs were equivalent to nurse-taken swabs and predicted persistent nasal carriage over four weeks.

## Introduction

*Staphylococcus aureus* is a widespread commensal organism, whose primary human reservoir is the nose.^1^ *S. aureus* nasal carriage (SANC) prevalence in adults is about 30% on cross-sectional studies.^2^ Carriage also occurs at other sites, and throat swabs are reported as more effective for detecting meticillin-resistant *S. aureus* (MRSA).^3^, ^4^, ^5^

Population genomics suggest that SANC is usually founded as a single colonizing event, but mixed carriage can be demonstrated in a minority using relatively low-resolution methods such as *spa* typing.^6^, ^7^ Moreover, SANC exhibits complex dynamics within individuals, with acquisition and loss of *S. aureus* occurring.^8^ Classifications of carriage states differ widely. A longitudinal study of SANC in 571 adults over two years defined carriage loss as two or more negative swabs two months apart.^8^ One classification defines ‘persistent’ carriers as isolation of *S. aureus* from >80% of cultures, and another uses qualitative and quantitative cultures to identify as ‘truly persistent’ and ‘never’ carriers.^9^, ^10^

Identifying and understanding *S. aureus* carriage is important in clinical settings because *S. aureus* causes infections ranging from superficial boils to life-threatening septicaemia, pneumonia, and osteomyelitis, and SANC increases the risk of these infections.^2^, ^11^, ^12^, ^13^, ^14^ Infection prevention strategies such as decolonization successfully reduce the incidence of postoperative infection in carriers, and screening allows the targeting of these interventions and reduced antimicrobial use.^15^, ^16^ Screening with targeted decolonization has been integrated into some postoperative infection prevention guidelines, but the design of programmes for screening and targeted decolonization remains an unsolved challenge.^17^, ^18^

Both research studies and pre-admission screening to target preoperative decolonization require either extensive investigator or healthcare personnel time, or reliance on self-swabbing. There is growing evidence from large studies that self-swabbing is acceptable to study participants, but there is limited data on the accuracy of self-swabbing by patients.^19^, ^20^ One study compared investigator to participant swabs, but these study participants were nursing personnel, and may not have represented patients who are not healthcare professionals.^21^ Further, it is unclear how accurately samples taken weeks before admission predict carriage at admission.^17^

This study addressed these uncertainties by assessing *S. aureus* carriage in healthy individuals, sampling this cohort every three to four days over four weeks. Nasal swabs underwent culture for *S. aureus* and all isolates underwent spa typing. Our aims were: (i) to compare isolation rates from three different body sites; (ii) to compare the results of nurse and participant swabs; (iii) to consider the ability of single nasal swab to predict persistent carriage over the next four weeks; and (iv) to identify new acquisitions using *spa* typing. Collectively this study aimed to improve our understanding of what can be predicted about carriage by a single nasal swab.

## Methods

Eligible participants were adults aged ≥16 years, who were invited when attending two general practices between October and December 2011 (Oxfordshire Ethics Committee B, reference 08/H0605/102). Written consent was obtained from all participants. The recruiting nurse took multi-site swabs from nose, axilla, and throat. Participants were trained to sample both anterior nares with a dry swab and given a leaflet demonstrating the technique. Packs with numbered swabs and return envelopes were provided and individuals took subsequent nose swabs themselves, returning them by mail to the John Radcliffe Hospital. Swabs were taken twice weekly for four weeks.

Swabs were returned in charcoal media within a week from sample collection and were stored at 4°C before processing (within four days). Swabs were incubated in 5% saline enrichment broth (Oxoid Ltd, Basingstoke, UK) overnight at 37°C before subculture on to SaSelect chromogenic agar (Bio-Rad Laboratories Ltd, Watford, UK) for 24 h. Confirmation of *S. aureus* isolates was by DNase and Prolex Staph Xtra Latex kit (Pro-Lab Diagnostics, Wirral, UK). Meticillin resistance was tested on Columbia agar with 5.0% salt (Oxoid Ltd) with BBL™ Sensi-Disc™ 1 μg Oxacillin discs (BD, Oxford, UK). A mixture of isolates taken from a sweep of the culture plate was stored at –80°C in 15% glycerol. Extraction of crude DNA and *spa* typing of isolates was performed as previously described with chromatograms analysed using the software Ridom StaphType v2.0.3 (Ridom GmbH, Münster, Germany).^22^ Where mixed chromatograms were found, *spa* typing was repeated using 12 individual colonies.

Carriage was defined as a nose, throat, or axilla swab positive for growth of *S. aureus.* Persistent carriage was assessed in those returning more than three self-swabs and was defined as the presence of *S. aureus* in all nose self-swabs returned in the study period, or at most one nose self-swab negative. Nasal carriage loss was defined as ≥2 consecutive post-baseline nasal swabs negative after previous nasal carriage detection. A single negative nose swab was not considered carriage loss, due to the limited sensitivity of swabbing.

Data were analysed using R (3.2.0). Proportions were compared using χ^2^-test or Fisher’s exact test (depending upon cell size).

## Results

**Figure 1:**
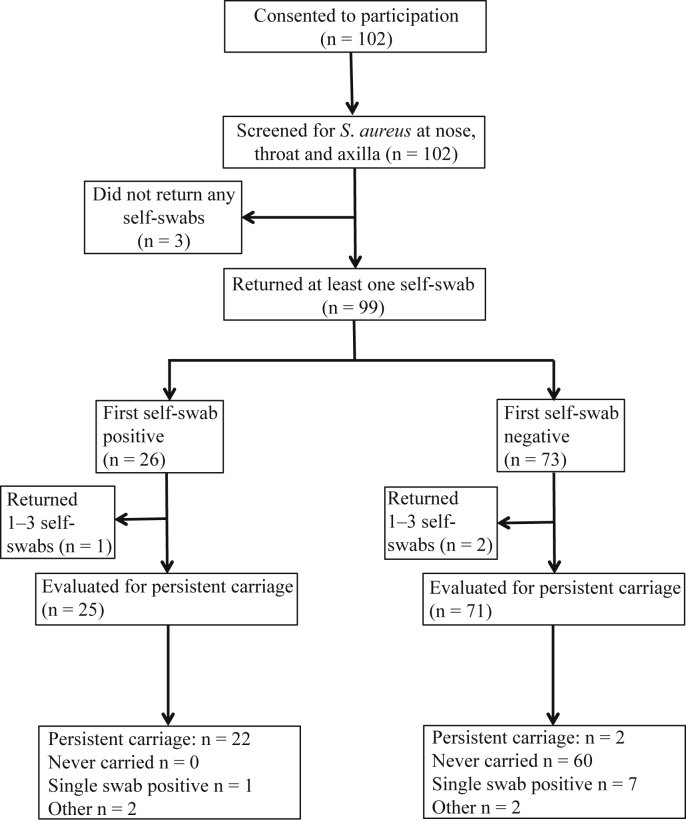
Flow chart of patient participation from recruitment, through nurse swabbing, first self-swab, and repeated self-swabs. Numbers of patients included and excluded at each stage are shown, along with the patterns detected on multiple self-swabs.

A total of 102 individuals [mean age: 60 years (SD: 15.2), 39% male] were recruited between October and December 2011. Of these, 99 returned at least one self-swab and 96 returned more than three self-swabs (Figure 1). The median time between nurse swab and arrival of first self-swab was six days.

### Multi-site swabbing by study personnel

**Figure 2:**
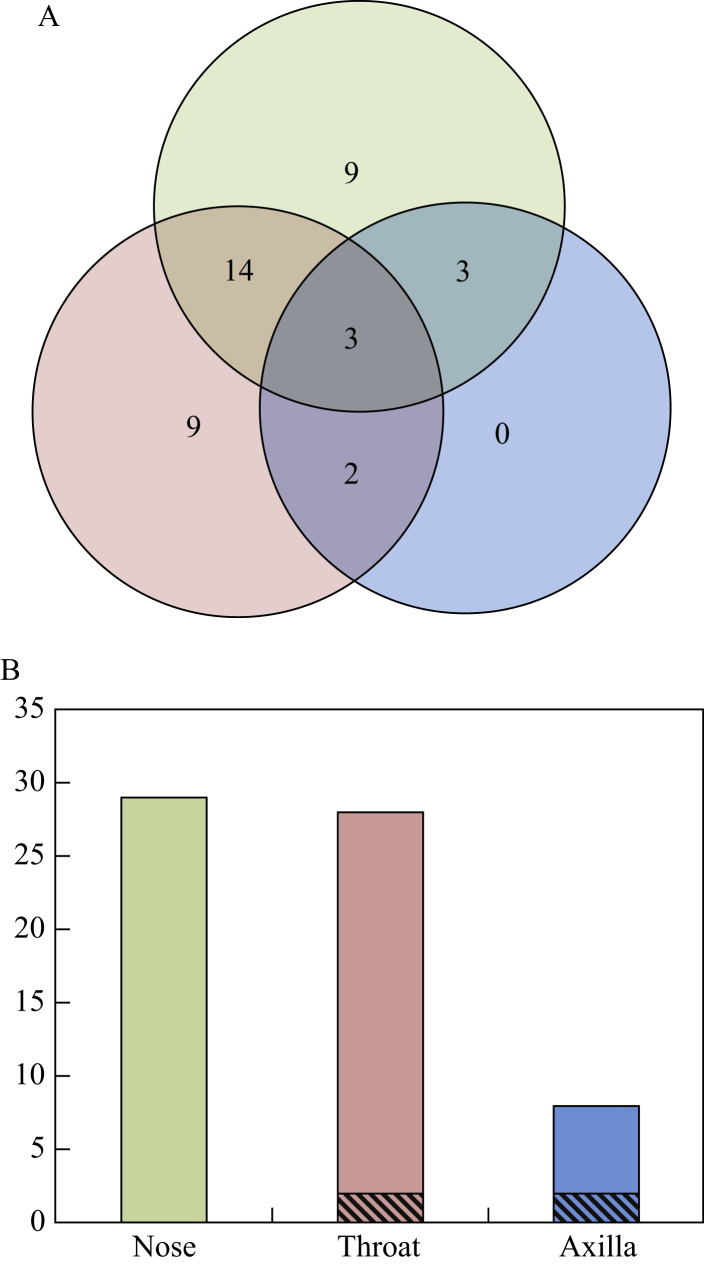
(A) A total of 102 patients underwent swabbing of three sites by a study nurse. Numbers in circles are the numbers of participants with *S. aureus* cultured from nose only (green), throat only (red) or axilla only (blue), and number of people with multiple *S. aureus* cultured from each combination of anatomical sites. (B) Number of patients with *S. aureus* cultured from a swab from each anatomical site with a single *spa* type (solid colours) or a mixture of *spa* types (hatched).

A total of 102 individuals underwent multi-site swabbing, and *S. aureus* carriage was detected from at least one site in 40 individuals, an overall carriage rate of 39%. There was no difference in the isolation rate between nose (29/102, 28%) and throat (28/102, 27%) (χ^2^: *P* = 0.88). Axilla swabs were positive in 8% (8/102), significantly lower than both nose (exact *P* = 0.0002) and throat (exact *P* = 0.0004). *S. aureus* was detected at multiple sites in 22 participants (22%) (Figure 2A). Whereas 18% of participants had only nose or throat carriage (9% each), there were no participants with carriage in the axilla only. All *S. aureus* isolates were meticillin susceptible.

All isolates underwent *spa* typing (Supplementary Table I). Of 22 individuals with carriage at multiple sites, 15 (68%) had identical *spa* profiles at all sites, and seven had discordant *spa* profiles (Table I). Three participants (5057, 5076, 5082) had a mixture at one site, with a matching single *spa* type at another site. Three participants (5001, 5031, 5046) had different *spa* types at two sites, with no overlap between sites. Participant 5067 had two *spa* types, with a different profile at each site (a mixture of two *spa* types in axilla, with each of the *spa* types found as single *spa* in the nose and throat). Axillary swabbing detected more mixed *spa* type carriage (Figure 2B), with the axillary swab significantly more likely to be mixed than nasal (2/8 mixed vs 0/29 mixed; exact *P* = 0.04).

**Table I:** *spa* types recovered in individuals with carriage at multiple sites and discordant *spa* profiles

Participant	Nose	Throat	Axilla
5001	t748	t9725	NG
5031	NG	t156	t748
5046	NG	t085	t008
5057	t3978	t3978/t688	NG
5067	t722	t954	t722/t954
5076	t385	NG	t385/t9974
5082	t2643	t2643/t521	NG

NG, *S. aureus* not grown at this site.

### Nose swabbing by participants

Ninety-nine returned at least one self-swab (Figure 1); for these 99, self-collected nasal swabs were compared with nurse-collected nasal and throat swabs. Nurse-administered nasal swabs were similar to the first self-administered swabs in terms of carriage rates [28/99 (28%) vs 26/99 (26%) respectively; χ^2^: *P* = 0.75]. Considering carriage on either swab as positive, sensitivity was similar for nurse-administered and self-swabs [28/31 (90%) versus 26/31 (84%), exact *P* = 0.71]. For those with nasal carriage found by both, *spa* types showed high concordance: 22/23 (96%) had identical *spa* typing results. One individual had a multiple *spa* types in the self-swab with only one of the two *spa* types detected on nurse nose swab. In contrast, whereas nurse-administered throat swabs showed similar sensitivity to self-collected nose swabs [both finding 26/33 (79%), exact *P* = 1], *spa* types were concordant in only 11/16 (69%), significantly less than that seen between nurse nose swab and self nose swab (exact *P* = 0.03). Two out of 16 (12%) individuals showed a mixture of spa types on nurse-collected throat swab not found on the self-swab, and 3/16 (19%) showed unrelated spa types on each.

### Twice weekly nose swabbing over one month

**Figure 3:**
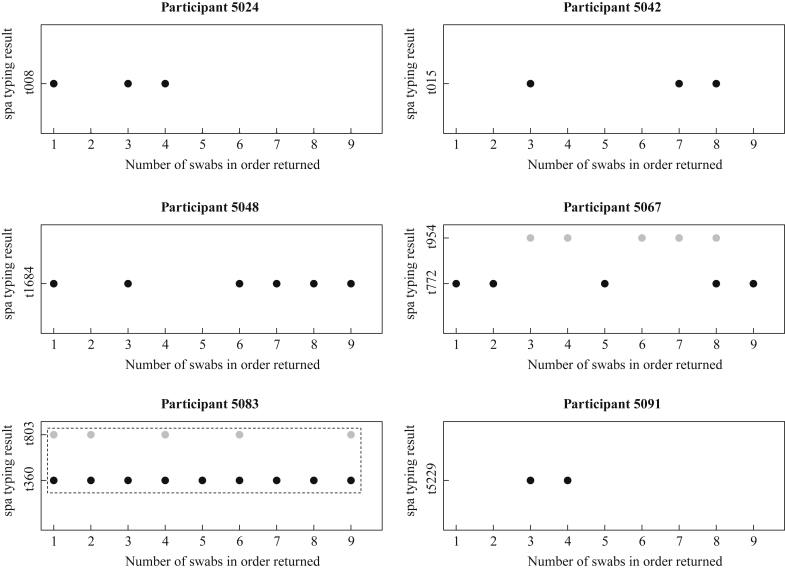
Ninety-nine patients returned self-swabs from the nose over a four-week period. Four participants were found to have carriage on multiple swabs without meeting the definition of persistent carriage and two showed changing *spa* type. *spa* types recovered are plotted against swab number; related *spa* types are enclosed in a dotted-line box.

In the 96 participants who returned more than three self-administered swabs, the first swab predicted persistent carriage over the next four weeks (Figure 1). Twenty-five had an initial positive swab, and, of these, 20 (80%) were positive on all swabs throughout the study period and two participants (5039 and 5086) had just one swab negative from a series of positive swabs. Of the remaining three: participant 5031 had only one swab positive, participant 5024 lost carriage at swab 5, and participant 5048 showed both carriage loss (swab 4) and gain (swab 6) (Figure 3, Supplementary Table I).

Of the 71 individuals evaluated for persistent carriage whose first swab was negative, 60 (85%) had negative swabs throughout the next four weeks. Of the remaining 11, seven participants (5032, 5035, 5036, 5054, 5062, 5093, 5100) had a single swab positive; two participants (5077, 5098) had only the first swab negative and were otherwise positive, meeting the definition for persistent carriage. Participant 5091 showed gain (swab 3) and loss (swab 5), and participant 5042 showed multiple gains (swab 3, swab 7) and loss (swab 4) (Figure 3, Supplementary Table I).

Twenty-two out of 25 participants with first swab positive were classified as persistent carriers over the next four weeks and 69/71 with first swab negative were not, giving a high positive predictive value (88%), and a very high negative predictive value (97%).

Twenty-eight participants returned more than three positive swabs; 24 were persistent carriers and four had multiple changes from positive to negative results with the same *spa* type on each positive swab (Supplementary Table I). Twenty-two of 24 persistent carriers showed a single *spa* type throughout the study, indicating stable *spa* type carriage. Two persistent carriers had variable *spa* typing profiles (Figure 3): participant 5083 carried a mixture of two related *spa* types (t360 and t803) on five swabs, with *spa* type t360 found alone on the other four swabs; participant 5067 had two unrelated *spa* types (t772 and t954) found on four swabs each with the *spa* profile switching five times and with a mixture of the two found on the penultimate swab. The carried *spa* type was stable during the study period for 26/28 (93%) individuals with *S. aureus* on multiple swabs; a small minority (2/28) displayed mixed and changing *spa* profiles.

## Discussion

Nasal swabs had the highest rate of detecting *S. aureus*, and did not differ significantly from throat swabs in the estimated rate of carriage. Other studies have identified higher rates of throat carriage; this may vary according to the study population.^4^, ^5^ Our results are consistent with other studies in which throat swabs detected around 10% additional carriers.^23^ Whereas no single site detects all *S. aureus* carriage, studies demonstrating that *S. aureus* carriage is a risk for infection have focused on SANC.^5^, ^11^, ^12^, ^13^, ^14^, ^15^, ^23^ This study supports the use of nasal swabs to detect *S. aureus* carriage, finding that throat swabbing is additive but not superior for detecting carriage, and that the *spa* types isolated from throat and nose show limited concordance.

In this study, *spa* typing indicated that the axilla swab was more likely to be mixed, but the clinical significance of this is unclear. Although the use of an axilla swab did not increase the detection of carriage, the greater diversity of *spa* types identified from this anatomical site suggests that the inclusion of axillary sampling may help detect carriers of a specific strain in outbreak investigations.

Self-administered swabs after education performed similarly well to nurse-administered swabs, adding to the existing evidence that self-swabbing is acceptable, and further indicating that it is a sensitive tool for larger epidemiological studies of SANC.^19^, ^21^ Several such studies have used self-administered swabs for cross-sectional and cohort studies, and this study, though limited in sample size, demonstrates the accuracy of self-swabbing by patients who are not healthcare professionals.^8^, ^19^, ^20^

Previous studies of SANC have conducted swabbing at intervals of one or two months, the latter study demonstrating that carriage gain and loss are frequent events.^8^, ^20^ Sampling nasal carriage more frequently, we demonstrated that an initial swab was highly predictive of carriage over the following month, complementing a previous finding that two positive nasal cultures a week apart predicted persistent carriage over 12 weeks.^10^ Our data also show that *spa* types were stable over the short term in most individuals. Both participants with changing *spa* types showed a mixture on at least one sample. These results may reflect mixed colonization with varying results due either to sampling or fluctuating population size. Alternatively they may represent repeated acquisitions from a close contact. These findings support the use of screening at four- to eight-week intervals in epidemiological studies of SANC dynamics.

Identifying SANC has several important implications in healthcare. Screening for MRSA has been the focus of infection control policies in order both to halt transmission and to prevent invasive infection.^16^, ^23^ For the prevention of invasive infection, detection of meticillin-susceptible *S. aureus* (MSSA) is also important, as MSSA is responsible for many serious infections.^24^ *S. aureus* infection is usually related to SANC, and evidence suggests that *S. aureus* undergoes genetic modification in the transition from carriage to invasion.^13^, ^14^, ^25^ Decolonization treatment can reduce infections originating from SANC: targeted decolonization reduces surgical site infection; non-targeted decolonization reduces all bacteraemias in an ICU setting, and in the following year outside the ICU.^15^, ^26^, ^27^ Outside of the ICU, non-targeted use of antibiotics is unlikely to be supported amid increasing recognition of the impact of antimicrobial resistance.^18^ Our study raises the possibility of patient self-swabbing for effective and resource-efficient pre-admission screening for SANC and targeted preoperative decolonization.

In conclusion, this study found that nose and throat nurse swabs identified similar overall rates of carriage, and that self-administered nasal swabs were a reliable method of identifying carriage and of predicting the likelihood of carriage over a four-week period.
