# Supplementary material for: Multi-site and nasal swabbing for carriage of Staphylococcus aureus: what does a single nose swab predict?
Source: J Hosp Infect. 2017 Jul;96(3):232–7. doi: 10.1016/j.jhin.2017.01.015 (PMC5490851; doi:10.1016/j.jhin.2017.01.015)
Supplement: Supplementary file 1 [file mmc1.docx]

| Classification | Participant | Nurse nasal swab | Swab 1 | Swab 2 | Swab 3 | Swab 4 | Swab 5 | Swab 6 | Swab 7 | Swab 8 | Swab 9 |
| --- | --- | --- | --- | --- | --- | --- | --- | --- | --- | --- | --- |
| Persistently positive (*N* = 24) | 5001 | t748 | t748 | t748 | t748 | t748 | t748 | t748 | t748 | t748 | t748 |
|  | 5002 | t209 | t209 | t209 | t209 | t209 | t209 | t209 | t209 | t209 | t209 |
|  | 5004 | t548 | t548 | t548 | t548 | t548 | t548 | t548 | t548 | t548 | t548 |
|  | 5011 | t002 | t002 | t002 | t002 | t002 | t002 | t002 | t002 | t002 | t002 |
|  | 5015 | t002 | t002 | t002 | t002 | t002 | t002 | t002 | t002 | t002 | t002 |
|  | 5016 | t939 | t939 | t939 | t939 | t939 | t939 | t939 | t939 | t939 | t939 |
|  | 5018 | t276 | t276 | t276 | t276 | t276 | t276 | t276 | t276 | t276 | t276 |
|  | 5030 | t6703 | t6703 | t6703 | t6703 | t6703 | t6703 | t6703 | t6703 | t6703 | t6703 |
|  | 5033 | t2538 | t2538 | t2538 | t2538 | t2538 | t2538 | t2538 | t2538 | t2538 | t2538 |
|  | 5039 | t002 | t002 | t002 | NG | t002 | t002 | t002 | t002 | t002 | t002 |
|  | 5051 | NG | t258 | t258 | t258 | t258 | t258 | t258 | t258 | t258 | t258 |
|  | 5057 | t3978 | t3978 | t3978 | t3978 | t3978 | t3978 | t3978 | t3978 | t3978 | X |
|  | 5066 | t2643 | t2643 | t2643 | t2643 | t2643 | t2643 | t2643 | t2643 | t2643 | t2643 |
|  | 5067 | t772 | t772 | t772 | t954 | t954 | t772 | t954 | t954 | t772/t954 | t772 |
|  | 5068 | t189 | t189 | t189 | t189 | t189 | t189 | t189 | t189 | t189 | t189 |
|  | 5077 | t012 | NG | t012 | t012 | t012 | t012 | t012 | t012 | t012 | t012 |
|  | 5082 | t2643 | t2643 | t2643 | t2643 | t2643 | t2643 | t2643 | t2643 | t2643 | t2643 |
|  | 5083 | t360 | t360/t803 | t360/t803 | t360 | t360/t803 | t360 | t360/t803 | t360 | t360 | t360/t803 |
|  | 5086 | t922 | t922 | t922 | NG | t922 | t922 | t922 | t922 | t922 | t922 |
|  | 5087 | t871 | t871 | t871 | t871 | t871 | t871 | t871 | t871 | t871 | t871 |
|  | 5089 | t9853 | t9853 | t9853 | t9853 | t9853 | t9853 | t9853 | t9853 | t9853 | t9853 |
|  | 5098 | t493 | NG | t493 | t493 | t493 | t493 | t493 | t493 | t493 | t493 |
|  | 5099 | t021 | t021 | t021 | t021 | t021 | t021 | t021 | t021 | t021 | t021 |
|  | 5102 | t471 | t471 | t471 | t471 | t471 | t471 | t471 | t471 | t471 | t471 |
| Single swab positive | 5031 | NG | t127 | NG | NG | NG | NG | NG | NG | NG | NG |
|  | 5032 | NG | NG | NG | t002 | NG | NG | NG | NG | NG | NG |
| (*N* = 9) | 5035 | NG | NG | NG | NG | NG | t1767 | NG | NG | NG | NG |
|  | 5036 | NG | NG | NG | t1996 | NG | NG | NG | NG | NG | NG |
|  | 5054 | NG | NG | NG | t9852 | NG | NG | NG | NG | NG | NG |
|  | 5062 | NG | NG | NG | NG | NG | NG | NG | NG | NG | NG |
|  | 5076 | t385 | NG | NG | NG | NG | NG | NG | NG | NG | NG |
|  | 5093 | NG | NG | NG | NG | NG | NG | NG | NG | NG | NG |
|  | 5100 | NG | NG | t4086 | NG | NG | NG | NG | NG | t177 | NG |
| Other (*N* = 4) | 5024 | NG | t008 | NG | t008 | t008 | NG | NG | NG | NG | NG |
|  | 5042 | NG | NG | NG | t015 | NG | NG | NG | t015 | t015 | NG |
|  | 5048 | t1684 | t1684 | NG | t1684 | NG | NG | t1684 | t1684 | t1684 | t1684 |
|  | 5091 | NG | NG | NG | t5229 | t5229 | NG | NG | NG | NG | NG |
| Not evaluable | 5005 | t9906 | NG | t9906 | X | X | X | X | X | X | X |
| (*N* = 4) | 5055 | t015 | t015 | t015 | X | X | X | X | X | X | X |
|  | 5095 | t127 | NG | NG | NG | X | X | X | X | X | X |
|  | 5096 | t002 | X | X | X | X | X | X | X | X | X |

**Supplementary Table I**

Results for all participants with at least one nasal swab growing *Staphylococcus aureus*.

**[footnote:]**

Swab number refers to the order in which swabs were received by the laboratory. Where *S. aureus* was isolate from nasal swab, all spa types identified are listed. NG indicates that *S. aureus* was not cultured from swab. X indicates that swab was not returned.
